# Relationship Between Non-fasting Triglycerides and Cardiovascular Disease Mortality in a 20-year Follow-up Study of a Japanese General Population: NIPPON DATA90

**DOI:** 10.2188/jea.JE20200399

**Published:** 2022-07-05

**Authors:** Aya Hirata, Tomonori Okamura, Takumi Hirata, Daisuke Sugiyama, Takayoshi Ohkubo, Nagako Okuda, Yoshikuni Kita, Takehito Hayakawa, Aya Kadota, Keiko Kondo, Katsuyuki Miura, Akira Okayama, Hirotsugu Ueshima

**Affiliations:** 1 Department of Preventive Medicine and Public Health, Keio University School of Medicine, Tokyo, Japan; 2 Department of Public Health, Hokkaido University Faculty of Medicine, Sapporo, Japan; 3 Faculty of Nursing and Medical Care, Keio University, Fujisawa, Kanagawa, Japan; 4 Department of Hygiene and Public Health, Teikyo University School of Medicine, Tokyo, Japan; 5 Department of Health and Nutrition, University of Human Arts and Sciences, Saitama, Japan; 6 Tsuruga City University of Nursing, Fukui, Japan; 7 Research Center for Social Studies of Health and Community, Ritsumeikan University, Kyoto, Japan; 8 Department of Public Health, Shiga University of Medical Science, Shiga, Japan; 9 Center for Epidemiologic Research in Asia, Shiga University of Medical Science, Shiga, Japan; 10 Research Institute of Strategy for Prevention, Tokyo, Japan

**Keywords:** non-fasting triglyceride, cardiovascular disease, mortality, general population, Japan

## Abstract

**Background:**

Non-fasting triglycerides (TG) are considered a better predictor of cardiovascular disease (CVD) than fasting TG. However, the effect of non-fasting TG on fatal CVD events remains unclear. In the present study, we aimed to explore the relationship between non-fasting TG and CVD mortality in a Japanese general population.

**Methods:**

A total of 6,831 participants without a history of CVD, in which those who had a blood sampling over 8 hours or more after a meal were excluded, were followed for 18.0 years. We divided participants into seven groups according to non-fasting TG levels: ≤59 mg/dL, 60–89 mg/dL, 90–119 mg/dL, 120–149 mg/dL, 150–179 mg/dL, 180–209 mg/dL, and ≥210 mg/dL, and estimated the multivariable-adjusted hazard ratios (HRs) of each TG group for CVD mortality after adjusting for potential confounders, including high density lipoprotein cholesterol. Additionally, we performed analysis stratified by age <65 and ≥65 years.

**Results:**

During the follow-up period, 433 deaths due to CVD were detected. Compared with a non-fasting TG of 150–179 mg/dL, non-fasting TG ≥210 mg/dL was significantly associated with increased risk for CVD mortality (HR 1.56: 95% CI, 1.01–2.41). Additionally, lower levels of non-fasting TG were also significantly associated with increased risk for fatal CVD. In participants aged ≥65 years, lower levels of non-fasting TG had a stronger impact on increased risk for CVD mortality, while higher levels of non-fasting TG had a stronger impact in those aged <65 years.

**Conclusion:**

In a general Japanese population, we observed a U-shaped association between non-fasting TG and fatal CVD events.

## INTRODUCTION

Research in most countries has traditionally observed triglycerides (TG) in the fasting state and, since the 1990s, a number of studies including meta-analyses have reported an association between higher levels of fasting TG and increased risk for ischemic cardiovascular events, even after adjusting for high-density lipoprotein cholesterol (HDL-C).^1^^–^^3^ Meanwhile, recent evidence suggests that, in addition to fasting TG, non-fasting TG levels may also have a marked impact on cardiovascular disease (CVD) risk.^4^^–^^8^ Two cohort studies, the Women’s Health Study in the United States and the Circulatory Risk in Communities Study in Japan, reported that non-fasting TG levels are a better predictor of CVD events.^5^^,^^6^ Additionally, the Copenhagen City Heart Study also showed a significant relationship between increased levels of non-fasting TG and the risk of atherosclerotic cardiovascular disease (ASCVD) events and all-cause mortality.^7^^,^^8^

TG levels gradually increase after a meal, taking 8 to 10 hours to reach a fasting state.^7^ Because we eat regularly during the day, the body spends most of its time in the non-fasting state. Additionally, levels of non-fasting TG strongly reflect levels of remnant lipoprotein, which contributes to the progression of atherosclerosis, compared with levels of fasting TG.^9^^–^^11^ This suggests that assessment of non-fasting TG levels may be important for the prevention of CVD. However, studies on the effect of non-fasting TG on CVD are limited. In particular, few studies have focused on fatal cardiovascular events, with most studies rather having focused mainly on non-fatal events; meanwhile, a previous study has reported higher levels of non-fasting TG were significantly associated with CVD mortality in the Norwegian Counties Study. However, the few studies on fatal events do not provide us any confirmation of the association between non-fasting TG and CVD mortality.

Accordingly, we explore the impact of non-fasting TG on CVD mortality in a Japanese general population.

## METHODS

### Study participants

NIPPON DATA 90 (National Integrated Project for Prospective Observation of Non-communicable Disease and Its Trends in the Aged 1990) is a cohort study based on the National Survey on Circulatory Disorders of Japan. The baseline surveys of NIPPON DATA90 were conducted in 1990. The details of these studies have been reported elsewhere.^12^^–^^14^ The present study was approved by the Institutional Review Board of Shiga University of Medical Science (No. 12–18, 2000; No. 17–21-1, 2010) and Keio University School of Medicine (2018-0108).

A total of 8,383 residents (3,504 men and 4,879 women, aged ≥30 years) were randomly selected from 300 districts in the survey area, and followed until November 15, 2010. The survey participation rate was 76.5%. Of the 8,383 participants, 1,552 were excluded for the following reasons: 248 had a history of cardiovascular disease, 190 reported intervals of over 8 hours or more between meals, 236 used medication for dyslipidemia, 628 had missing data at baseline, and 250 were lost to follow-up because of incomplete residential access information. Finally, 6,831 participants (2,853 men and 3,978 women) were included in the analysis.

### Baseline examination

The baseline survey measured height, weight, and blood pressure, performed blood tests, and administered a questionnaire on lifestyle. Non-fasting blood samples were obtained from all participants and shipped to a central laboratory (SRL, Tokyo, Japan) for analysis. Blood samples were collected at the following time intervals after meals: <0.5 h (5.8%), 0.5 to <1 h (5.7%), 1 to <2 h (10.0%), 2 to <3 h (20.9%), 3 to <4 h (18.9%), 4 to <6 h (30.2%), 6 to <8 h (8.5%). Serum total cholesterol (TC) and non-fasting TG were measured using enzymatic methods. HDL-C was measured using precipitation methods with heparin-calcium. Lipid measurement was standardized according to the Centers for Disease Control/National Heart, Lung, and Blood Institute Lipid Standardization Program.^15^ Glycated hemoglobin (HbA1c; Japan Diabetes Society [JDS]) values were converted to HbA1c (National Glycohemoglobin Standardization Program [NGSP]) values using the following formula proved by JDS: HbA1c NGSP value (%) = 1.02 × JDS value (%) + 0.25.^16^ HbA1c (NGSP) values were used in the present analyses. Plasma glucose level was measured enzymatically.

Public health nurses obtained lifestyle information, such as smoking status; alcohol drinking status; use of medication for dyslipidemia, hypertension, and diabetes; and history of CVD. Body mass index (BMI) was calculated as the weight (kg) divided by the height squared (m^2^). Baseline blood pressure was measured by trained observers using a standard mercury sphygmomanometer on the right arm of seated participants.

We defined hypertension as systolic blood pressure (SBP) ≥140 mm Hg, diastolic blood pressure (DBP) ≥90 mm Hg, or taking medicine for hypertension; and diabetes as non-fasting blood glucose ≥200 mg/dL, HbA1c (NGSP) ≥6.5%, or taking medicine for diabetes. Smoking status and alcohol drinking status were each categorized as never, past, and current users.

### Follow-up survey

We obtained information on deaths at the follow-up survey. Underlying causes of death listed in the National Vital Statistics were coded according to the International Classification of Diseases (ICD)-9 through the end of 1994, and according to ICD-10 from the start of 1995 through the end of 2010. The codes were defined as follows: CVD, from 393 to 459 (ICD-9) and from I00 to I99 (ICD-10); coronary heart disease (CHD), from 410 to 414 (ICD-9) and from I20 to I25 (ICD-10); cerebral infarction, from 430 to 438 (ICD-9) and from I60 to I69 (ICD-10); cerebral infarction, 433, 434, 437.8a, and 437.8b (ICD-9) and I63 and I69.3 (ICD-10); ASCVD (defined as CHD and cerebral infarction); cerebral hemorrhage, from 431 to 432 (ICD-9) and I61 and I69.1 (ICD-10). Details of these classifications were described previously.^12^^–^^14^

### Statistical analysis

We divided the participants into seven groups according to non-fasting TG levels: ≤59 mg/dL, 60–89 mg/dL, 90–119 mg/dL, 120–149 mg/dL, 150–179 mg/dL, 180–209 mg/dL, and ≥210 mg/dL. Mean values and standard deviation (SD) were reported for continuous variables with a normal distribution, median and interquartile range for those with a skewed distribution, and number and proportion for categorical variables by non-fasting TG category. A Cox proportional hazards model was used to estimate the age- and multivariable-adjusted hazard ratio (HR) of each non-fasting TG category for all-cause death, total CVD death, cause-specific CVD death, and non-CVD mortality compared with a TG of 150–179 mg/dL. We determined a TG of 150–179 mg/dL as a reference because hazard ratios of non-fasting TG: 146–176 mg/dL were lowest among those for CVD mortality and ASCVD mortality according to decile category of non-fasting TG (eTable 1 and eTable 2). Model 1 was adjusted for age; model 2 was adjusted for the variables in model 1 plus BMI, total cholesterol (TC), hypertension, diabetes, smoking status, and alcohol drinking status; and model 3 was adjusted for the variables in model 2 plus HDL-C. The proportional hazard assumption was confirmed using the statistical test and graphical diagnostics based on the Schoenfeld residuals. Additionally, we performed the same analyses excluding deaths within first five years of follow-up (*n* = 256). Furthermore, we stratified participants by age <65 and ≥65 years and estimated the adjusted HR of each non-fasting TG category for CVD morality and ASCVD mortality in each age stratum. We generated an interaction term by multiplying non-fasting TG levels (continuous variable) and age group (<65 and ≥65 years) and analyzed the interaction with CVD mortality and ASCVD mortality using a Cox proportional hazards model, with adjustment for the variables in model 3. The interaction between non-fasting TG levels (continuous variable) and sex was also examined.

Furthermore, we evaluated the association between non-fasting TG levels and CVD mortality in all participants and those aged <65 and ≥65 years using restricted cubic splines incorporated in a Cox proportional hazards model. We decided a non-fasting TG of 150 mg/dL as the reference and 50, 100, 150, and 200 mg/dL as knots with restricted range of non-fasting TG levels ≤300 mg/dL. Confidence intervals (CIs) were estimated at the 95% level, and two-sided *P*-values <0.05 were considered significant. Statistical analysis was performed using STATA/SE 15 data analysis and statistical software (Stata Corp LP, College Station, TX, USA).

## RESULTS

Characteristics of the study participants according to non-fasting TG categories by sex at the baseline survey are shown in Table 1, and the overall results are shown in Table 2. Mean age was 52.8 years for men, 51.7 years for women, and 52.2 years overall. Mean age was lower among men and higher among women with increased levels of non-fasting TG. BMI, TC level, proportion of current smokers, proportion of current drinkers, prevalence of diabetes, and prevalence of hypertension were higher among both men and women with increased levels of non-fasting TG. Median and interquartile range according to time since the last meal and the distribution of time since the last meal according to non fasting triglyceride category were shown in eTable 3 and eTable 4, respectively.

**Table 1.** Characteristics of study participants according to non-fasting triglyceride category by sex at the baseline survey

	Baseline triglyceride level						
	≤59 mg/dL	60–89 mg/dL	90–119 mg/dL	120–149 mg/dL	150–179 mg/dL	180–209 mg/dL	≥210 mg/dL
**Men (*n* = 2,853)**							
Number of participants, *n*	254	587	595	398	295	213	511
Age, years	55.7 (14.3)	54.4 (14.4)	53.3 (13.3)	52.9 (13.1)	50.8 (12.2)	51.7 (13.5)	50.7 (12.6)
Body mass index, kg/m^2^	20.7 (2.6)	21.7 (2.6)	22.5 (3.0)	23.3 (2.8)	23.8 (2.8)	24.2 (2.8)	24.6 (2.8)
Non-fasting blood glucose, mg/dL	96.8 (26.5)	101.4 (27.6)	101.4 (27.1)	104.6 (44.2)	102.1 (31.7)	111.8 (50.2)	105.6 (37.7)
Hemoglobin A1c, %	5.3 (0.7)	5.3 (0.6)	5.3 (0.6)	5.4 (0.9)	5.3 (0.6)	5.5 (1.1)	5.5 (0.9)
Diabetes, *n* (%)	12 (4.7%)	21 (3.6%)	25 (4.2%)	33 (8.3%)	11 (3.7%)	20 (9.4%)	42 (8.2%)
Systolic blood pressure, mm Hg	136.1 (23.7)	136.6 (20.5)	137.1 (19.7)	138.5 (20.9)	135.7 (18.5)	139.4 (20.5)	139.3 (17.9)
Diastolic blood pressure, mm Hg	80.5 (12.5)	82.0 (11.5)	83.0 (11.5)	84.2 (11.7)	83.9 (10.7)	84.5 (12.5)	86.0 (11.1)
Hypertension, *n* (%)	102 (40.2%)	263 (44.8%)	292 (49.1%)	208 (52.3%)	138 (46.8%)	109 (51.2%)	284 (55.6%)
Total cholesterol, mg/dL	173.1 (28.2)	185.8 (29.8)	190.8 (32.3)	203.4 (33.7)	206.1 (33.1)	213.5 (40.9)	218.0 (39.3)
HDL cholesterol, mg/dL	60.9 (15.6)	57.1 (15.3)	52.7 (13.6)	49.7 (13.2)	45.5 (12.1)	44.7 (12.6)	40.5 (11.3)
Triglycerides, mg/dL	51 [46–55]	75 [68–83]	104 [97–111]	134 [127–142]	163 [156–170]	193 [186–200]	273 [237–345]
Current smoker, *n* (%)	140 (55.1%)	307 (52.3%)	335 (56.3%)	223 (56.0%)	181 (61.4%)	127 (59.6%)	291 (57.0%)
Current drinker, *n* (%)	148 (58.3%)	337 (57.4%)	349 (58.7%)	241 (60.6%)	166 (56.3%)	133 (62.4%)	319 (62.4%)

**Women (*n* = 3,978)**							
Number of participants, *n*	624	1,054	832	550	336	210	372
Age, years	45.2 (12.3)	49.4 (13.5)	53.1 (14.0)	54.0 (13.1)	56.2 (13.4)	56.2 (13.7)	56.5 (12.2)
Body mass index, kg/m^2^	21.2 (2.7)	22.0 (3.0)	22.9 (3.2)	23.5 (3.5)	24.1 (3.3)	24.0 (3.1)	24.5 (3.0)
Non-fasting blood glucose, mg/dL	93.9 (16.2)	97.8 (20.5)	102.6 (24.7)	106.1 (38.0)	105.2 (26.4)	107.9 (32.3)	114.9 (46.4)
Hemoglobin A1c, %	5.0 (0.4)	5.1 (0.6)	5.2 (0.6)	5.3 (0.7)	5.4 (0.9)	5.4 (0.7)	5.6 (1.1)
Diabetes, *n* (%)	6 (1.0%)	14 (1.3%)	25 (3.0%)	26 (4.7%)	22 (6.6%)	14 (6.7%)	39 (10.5%)
Systolic blood pressure, mm Hg	123.9 (17.7)	129.0 (19.3)	133.4 (20.2)	138.4 (22.3)	139.4 (20.4)	137.5 (19.4)	141.2 (20.3)
Diastolic blood pressure, mm Hg	75.3 (10.1)	77.6 (11.3)	79.6 (11.2)	80.8 (12.9)	83.3 (10.7)	81.0 (11.6)	83.4 (12.3)
Hypertension, *n* (%)	143 (22.9%)	335 (31.8%)	357 (42.9%)	285 (51.8%)	182 (54.2%)	114 (54.3%)	221 (59.4%)
Total cholesterol, mg/dL	184.0 (31.3)	196.9 (34.0)	204.9 (35.3)	214.4 (33.7)	219.4 (35.8)	220.1 (35.3)	231.3 (40.8)
HDL cholesterol, mg/dL	66.2 (13.8)	62.3 (14.0)	56.9 (13.2)	53.4 (12.0)	50.3 (12.2)	48.0 (11.1)	43.4 (11.3)
Triglycerides, mg/dL	49 [43–55]	75 [68–81]	104 [97–111]	132 [126–140]	164 [157–171]	194 [186–201]	256 [228–317]
Current smoker, *n* (%)	56 (9.0%)	76 (7.2%)	76 (9.1%)	51 (9.3%)	32 (9.5%)	24 (11.4%)	37 (10.0%)
Current drinker, *n* (%)	59 (9.5%)	72 (6.8%)	56 (6.7%)	28 (5.1%)	13 (3.9%)	14 (6.7%)	19 (5.1%)

HDL, high-density lipoprotein.

Data are presented as mean (standard deviation), median [interquartile range] or as a number (%).

**Table 2.** Characteristics of study participants according to non-fasting triglyceride category at the baseline survey

	Baseline triglyceride level						
	≤59 mg/dL	60–89 mg/dL	90–119 mg/dL	120–149 mg/dL	150–179 mg/dL	180–209 mg/dL	≥210 mg/dL
**Total (*n* = 6,831)**							
Number of participants, *n*	878	1,641	1,427	948	631	423	883
Age, years	48.2 (13.7)	51.2 (14.0)	53.2 (13.7)	53.5 (13.1)	53.7 (13.1)	53.9 (13.8)	53.1 (12.8)
Body mass index, kg/m^2^	21.1 (2.7)	21.9 (2.9)	22.7 (3.1)	23.4 (3.2)	24.0 (3.1)	24.1 (2.9)	24.6 (2.99
Non-fasting blood glucose, mg/dL	94.8 (19.8)	99.1 (23.4)	102.1 (25.7)	105.5 (40.7)	103.8 (29.0)	109.9 (42.3)	109.5 (41.8)
Hemoglobin A1c, %	5.1 (0.5)	5.2 (0.6)	5.2 (0.6)	5.3 (0.8)	5.4 (0.8)	5.5 (0.9)	5.5 (1.0)
Diabetes, *n* (%)	18 (2.1%)	35 (2.1%)	50 (3.5%)	59 (6.2%)	33 (5.2%)	34 (8.0%)	81 (9.2%)
Systolic blood pressure, mm Hg	127.4 (20.4)	131.7 (20.1)	134.9 (20.0)	138.5 (21.7)	137.7 (19.6)	138.5 (20.0)	140.1 (19.0)
Diastolic blood pressure, mm Hg	76.8 (11.1)	79.2 (11.6)	81.0 (11.4)	82.3 (12.5)	83.6 (10.7)	82.8 (12.2)	84.9 (11.7)
Hypertension, *n* (%)	245 (27.9%)	598 (36.4%)	649 (45.5%)	493 (52.0%)	320 (50.7%)	223 (52.7%)	505 (57.2%)
Total cholesterol, mg/dL	180.9 (30.8)	192.9 (33.0)	199.0 (34.8)	209.8 (34.1)	213.2 (35.1)	216.8 (38.4)	223.6 (40.5)
HDL cholesterol, mg/dL	64.7 (14.5)	60.4 (14.7)	55.1 (13.5)	51.9 (12.7)	48.1 (12.4)	46.4 (12.0)	41.7 (11.4)
Triglycerides, mg/dL	50 [44–55]	75 [68–82]	104 [97–111]	133 [126–140]	163 [156–170]	193 [186–201]	264 [231–336]
Current smoker, *n* (%)	196 (22.3%)	383 (23.3%)	411 (28.8%)	274 (28.9%)	213 (33.8%)	151 (35.7%)	328 (37.2%)
Current drinker, *n* (%)	207 (23.6%)	409 (24.9%)	405 (28.4%)	269 (28.4%)	179 (28.4%)	147 (34.8%)	338 (38.3%)

HDL, high-density lipoprotein.

Data are presented as mean (standard deviation), median [interquartile range] or as a number (%).

Total follow-up period was 49,750 person-years for men, 73,433 person-years for women, and 123,183 person-years overall. The mean follow-up period was 17.4 years for men, 18.4 years for women, and 18.0 years overall. During the follow-up period, 1,552 all-cause deaths were detected, 433 deaths due to CVD, 195 deaths due to ASCVD, 90 deaths due to CHD, 172 deaths due to stroke, 105 deaths due to cerebral infarction, and 40 deaths due to cerebral hemorrhage.

The crude mortality rates and adjusted HRs for CVD mortality, ASCVD mortality, CHD mortality, and cerebral infarction mortality for each non-fasting TG category are shown in Table 3 and Table 4. Compared with a non-fasting TG of 150–179 mg/dL, non-fasting TG ≥210 mg/dL was significantly associated with increased risk for CVD mortality among men (HR 2.38; 95% CI, 1.14–4.94) and all participants combined (HR 1.56; 95% CI, 1.01–2.41), but not among women (HR 1.20; 95% CI, 0.68–2.14). Additionally, lower levels of non-fasting TG were also significantly associated with increased risk for CVD mortality among men and all participants combined. These findings remained almost unchanged after excluding deaths within first 5 years of follow-up (data not shown). For ASCVD mortality, non-fasting TG ≥180 mg/dL was associated with an increased but non-statistically significant risk, while non-fasting TG ≤59 mg/dL was significantly associated with increased risk for ASCVD mortality among men (HR 4.47; 95% CI, 1.46–13.6) and all participants combined (HR 2.29; 95% CI, 1.10–4.78). No interaction was observed between non-fasting TG levels and sex for CVD mortality and ASCVD mortality. Regarding CHD death, non-fasting TG ≤59 mg/dL was significantly associated with an increased risk for ASCVD mortality among men (HR 5.37; 95% CI, 1.20–23.95) and all participants combined (HR 5.86; 95% CI, 1.73–19.78). In contrast, non-fasting TG level was not significantly associated with death due to cerebral infarction.

**Table 3.** Crude mortality rates and hazard ratios for CVD mortality and ASCVD mortality according to non-fasting triglyceride category

	Baseline non-fasting triglyceride level						
	≤59 mg/dL	60–89 mg/dL	90–119 mg/dL	120–149 mg/dL	150–179 mg/dL	180–209 mg/dL	≥210 mg/dL
**CVD death**							
Men							
Number of participants	254	587	595	398	295	213	511
Person-years	4,093	9,927	10,412	7,033	5,326	3,746	9,213
Number of deaths	24	52	47	26	9	16	40
Crude mortality rate	5.9	5.2	4.5	3.7	1.7	4.3	4.3
Hazard ratio (95% CI)							
Model 1	2.44 (1.13–5.27)	2.16 (1.06–4.39)	2.15 (1.05–4.39)	1.78 (0.83–3.80)	Ref.	2.24 (0.98–5.07)	2.53 (1.22–5.21)
Model 2	2.68 (1.20–5.97)	2.31 (1.12–4.79)	2.10 (1.02–4.32)	1.66 (0.77–3.56)	Ref.	2.16 (0.95–4.91)	2.50 (1.20–5.17)
Model 3	3.15 (1.38–7.20)	2.62 (1.24–5.50)	2.29 (1.10–4.76)	1.76 (0.82–3.78)	Ref.	2.19 (0.96–4.97)	2.38 (1.14–4.94)

Women							
Number of participants	624	1,054	832	550	336	210	372
Person-years	11,810	19,645	15,080	10,106	6,102	3,844	6,846
Number of deaths	22	49	54	31	22	13	28
Crude mortality rate	1.9	2.5	3.6	3.1	3.6	3.4	4.1
Hazard ratio (95% CI)							
Model 1	1.67 (0.92–3.03)	1.20 (0.72–1.99)	1.28 (0.78–2.11)	1.07 (0.61–1.85)	Ref.	0.84 (0.42–1.67)	1.18 (0.67–2.07)
Model 2	1.66 (0.89–3.08)	1.20 (0.72–2.02)	1.30 (0.78–2.15)	1.08 (0.62–1.88)	Ref.	0.76 (0.38–1.52)	1.20 (0.68–2.10)
Model 3	1.65 (0.86–3.14)	1.20 (0.70–2.05)	1.29 (0.78–2.16)	1.08 (0.62–1.88)	Ref.	0.76 (0.38–1.52)	1.20 (0.68–2.14)

Total							
Number of participants	878	1,641	1,427	948	631	423	883
Person-years	15,903	29,572	25,492	17,139	11,428	7,590	16,059
Number of deaths	46	101	101	57	31	29	68
Crude mortality rate	2.9	3.4	4.0	3.3	2.7	3.8	4.2
Hazard ratio (95% CI)							
Model 1	1.78 (1.13–2.81)	1.44 (0.96–2.16)	1.52 (1.01–2.28)	1.25 (0.80–1.94)	Ref.	1.25 (0.75–2.08)	1.60 (1.04–2.44)
Model 2	1.88 (1.16–3.04)	1.52 (1.00–2.30)	1.53 (1.02–2.30)	1.23 (0.79–1.91)	Ref.	1.16 (0.70–1.93)	1.62 (1.05–2.48)
Model 3	2.02 (1.23–3.32)	1.61 (1.05–2.47)	1.59 (1.05–2.40)	1.25 (0.81–1.95)	Ref.	1.16 (0.69–1.92)	1.56 (1.01–2.41)

**ASCVD death**							
Men							
Number of participants	254	587	595	398	295	213	511
Person-years	4,093	9,927	10,412	7,033	5,326	3,746	9,213
Number of deaths	14	26	23	12	5	7	18
Crude mortality rate	3.4	2.6	2.2	1.7	0.9	1.9	2.0
Hazard ratio (95% CI)							
Model 1	2.50 (0.90–6.98)	1.88 (0.71–4.91)	1.84 (0.70–4.86)	1.44 (0.50–4.10)	Ref.	1.71 (0.54–5.41)	2.01 (0.74–5.43)
Model 2	3.65 (1.24–10.72)	2.42 (0.90–6.51)	2.03 (0.76–5.40)	1.43 (0.50–4.09)	Ref.	1.64 (0.51–5.19)	1.85 (0.68–5.03)
Model 3	4.47 (1.46–13.6)	2.83 (1.03–7.78)	2.26 (0.84–6.09)	1.53 (0.53–4.39)	Ref.	1.66 (0.52–5.25)	1.73 (0.63–4.74)

Women							
Number of participants	624	1,054	832	550	336	210	372
Person-years	11,810	19,645	15,080	10,106	6,102	3,844	6,846
Number of deaths	8	21	19	14	9	6	13
Crude mortality rate	0.7	1.1	1.3	1.4	1.5	1.6	1.9
Hazard ratio (95% CI)							
Model 1	1.55 (0.60–4.05)	1.26 (0.57–2.75)	1.09 (0.49–2.42)	1.19 (0.51–2.76)	Ref.	0.94 (0.33–2.64)	1.37 (0.58–3.22)
Model 2	1.26 (0.46–3.44)	1.10 (0.49–2.46)	1.02 (0.45–2.29)	1.15 (0.49–2.69)	Ref.	0.90 (0.31–2.55)	1.37 (0.58–3.25)
Model 3	1.28 (0.45–3.62)	1.12 (0.49–2.58)	1.03 (0.45–2.33)	1.16 (0.49–2.70)	Ref.	0.90 (0.31–2.54)	1.35 (0.56–3.27)

Total							
Number of participants	878	1,641	1,427	948	631	423	883
Person-years	15,903	29,572	25,492	17,139	11,428	7,590	16,059
Number of deaths	22	47	42	26	14	13	31
Crude mortality rate	1.4	1.6	1.6	1.5	1.2	1.7	1.9
Hazard ratio (95% CI)							
Model 1	1.85 (0.94–3.63)	1.44 (0.79–2.63)	1.37 (0.74–2.51)	1.24 (0.64–2.38)	Ref.	1.21 (0.56–2.57)	1.58 (0.84–2.98)
Model 2	2.03 (1.00–4.13)	1.54 (0.83–2.86)	1.41 (0.76–2.60)	1.25 (0.65–2.41)	Ref.	1.15 (0.54–2.46)	1.57 (0.83–2.96)
Model 3	2.29 (1.10–4.78)	1.70 (0.90–3.21)	1.50 (0.80–2.79)	1.30 (0.67–2.50)	Ref.	1.14 (0.53–2.43)	1.48 (0.77–2.81)

CI, confidence interval; CVD, cardiovascular disease; ASCVD, atherosclerotic cardiovascular disease.

Model 1 was adjusted for age.

Model 2 was adjusted for variables in model 1 plus body mass index, total cholesterol, hypertension, diabetes, smoking status, and alcohol drinking status.

Model 3 was adjusted for variables in model 2 plus high-density lipoprotein cholesterol.

The model for total participants (in which sexes were combined) was also adjusted for sex.

Crude mortality rate is shown per 1,000 person-years.

**Table 4.** Crude mortality rates and hazard ratios for CHD mortality and cerebral infarction mortality according to non-fasting triglyceride category

	Baseline non-fasting triglyceride level						
	≤59 mg/dL	60–89 mg/dL	90–119 mg/dL	120–149 mg/dL	150–179 mg/dL	180–209 mg/dL	≥210 mg/dL
**CHD death**							
Men							
Number of participants	254	587	595	398	295	213	511
Person-years	4,093	9,927	10,412	7,033	5,326	3,746	9,213
Number of deaths	7	11	12	8	3	4	9
Crude mortality rate	1.7	1.1	1.2	1.1	0.6	1.1	1.0
Hazard ratio (95% CI)							
Model 1	2.28 (0.58–8.87)	1.48 (0.41–5.32)	1.72 (0.48–6.12)	1.72 (0.45–6.49)	Ref.	1.75 (0.39–7.86)	1.73 (0.46–6.41)
Model 2	3.31 (0.79–13.88)	1.78 (0.48–6.67)	1.81 (0.50–6.53)	1.66 (0.43–6.30)	Ref.	1.72 (0.38–7.72)	1.49 (0.39–5.59)
Model 3	5.37 (1.20–23.95)	2.55 (0.66–9.86)	2.34 (0.63–8.58)	1.95 (0.51–7.47)	Ref.	1.75 (0.38–7.85)	1.28 (0.33–4.84)

Women							
Number of participants	624	1,054	832	550	336	210	372
Person-years	11,810	19,645	15,080	10,106	6,102	3,844	6,846
Number of deaths	5	10	3	8	1	2	7
Crude mortality rate	0.4	0.5	0.2	0.8	0.2	0.5	1.0
Hazard ratio (95% CI)							
Model 1	8.51 (0.98–73.23)	5.31 (0.68–41.51)	1.57 (0.16–15.17)	6.17 (0.77–49.40)	Ref.	2.86 (0.25–31.62)	6.34 (0.77–51.58)
Model 2	8.51 (0.93–77.14)	5.20 (0.65–41.47)	1.56 (0.16–15.23)	6.42 (0.79–51.79)	Ref.	2.70 (0.24–29.89)	5.78 (0.70–47.34)
Model 3	9.00 (0.95–85.32)	5.44 (0.66–44.77)	1.60 (0.16–15.77)	6.49 (0.80–52.42)	Ref.	2.67 (0.24–29.62)	5.58 (0.66–46.53)

Total							
Number of participants	878	1,641	1,427	948	631	423	883
Person-years	15,903	29,572	25,492	17,139	11,428	7,590	16,059
Number of deaths	12	21	15	16	4	6	16
Crude mortality rate	0.8	0.7	0.6	0.9	0.4	0.8	1.0
Hazard ratio (95% CI)							
Model 1	3.41 (1.10–10.61)	2.30 (0.79–6.73)	1.74 (0.57–5.24)	2.69 (0.90–8.07)	Ref.	2.04 (0.57–7.23)	2.76 (0.92–8.28)
Model 2	4.28 (1.31–13.95)	2.61 (0.87–7.79)	1.83 (0.60–5.57)	2.68 (0.89–8.06)	Ref.	1.97 (0.55–6.99)	2.52 (0.83–7.60)
Model 3	5.86 (1.73–19.78)	3.34 (1.09–10.18)	2.14 (0.69–6.58)	2.93 (0.97–8.84)	Ref.	1.92 (0.54–6.82)	2.20 (0.72–6.68)

**Cerebral infarction death**							
Men							
Number of participants	254	587	595	398	295	213	511
Person-years	4,093	9,927	10,412	7,033	5,326	3,746	9,213
Number of deaths	7	15	11	4	2	3	9
Crude mortality rate	1.7	1.5	1.1	0.6	0.4	0.8	1.0
Hazard ratio (95% CI)							
Model 1	2.80 (0.57–13.56)	2.32 (0.52–10.23)	1.97 (0.43–8.93)	1.07 (0.19–5.87)	Ref.	1.61 (0.26–9.72)	2.36 (0.51–10.96)
Model 2	4.37 (0.84–22.75)	3.53 (0.77–16.20)	2.42 (0.52–11.16)	1.17 (0.21–6.44)	Ref.	1.53 (0.25–9.29)	2.45 (0.52–11.51)
Model 3	4.10 (0.75–22.25)	3.35 (0.70–15.86)	2.34 (0.50–10.93)	1.14 (0.20–6.34)	Ref.	1.53 (0.25–9.28)	2.51 (0.53–11.86)

Women							
Number of participants	624	1,054	832	550	336	210	372
Person-years	11,810	19,645	15,080	10,106	6,102	3,844	6,846
Number of deaths	3	11	16	6	8	4	6
Crude mortality rate	0.3	0.6	1.1	0.6	1.3	1.0	0.9
Hazard ratio (95% CI)							
Model 1	0.65 (0.17–2.46)	0.75 (0.30–1.87)	1.01 (0.43–2.38)	0.57 (0.19–1.65)	Ref.	0.69 (0.21–2.32)	0.75 (0.26–2.17)
Model 2	0.46 (0.11–1.85)	0.61 (0.24–1.57)	0.95 (0.39–2.26)	0.52 (0.18–1.53)	Ref.	0.70 (0.21–2.38)	0.81 (0.27–2.37)
Model 3	0.46 (0.11–1.95)	0.62 (0.23–1.67)	0.95 (0.39–2.31)	0.53 (0.18–1.54)	Ref.	0.70 (0.20–2.38)	0.80 (0.26–2.42)

Total							
Number of participants	878	1,641	1,427	948	631	423	883
Person-years	15,903	29,572	25,492	17,139	11,428	7,590	16,059
Number of deaths	10	26	27	10	10	7	15
Crude mortality rate	0.6	0.9	1.1	0.6	0.9	0.9	0.9
Hazard ratio (95% CI)							
Model 1	1.20 (0.49–2.90)	1.09 (0.52–2.27)	1.20 (0.58–2.49)	0.65 (0.27–1.58)	Ref.	0.86 (0.32–2.27)	1.11 (0.49–2.48)
Model 2	1.16 (0.45–2.96)	1.09 (0.51–2.34)	1.21 (0.57–2.54)	0.67 (0.27–1.62)	Ref.	0.82 (0.31–2.17)	1.16 (0.52–2.61)
Model 3	1.12 (0.42–2.95)	1.06 (0.48–2.33)	1.19 (0.56–2.52)	0.66 (0.27–1.61)	Ref.	0.82 (0.31–2.17)	1.18 (0.52–2.69)

CI, confidence interval; CHD, coronary heart disease.

Model 1 was adjusted for age.

Model 2 was adjusted for variables in model 1 plus body mass index, total cholesterol, hypertension, diabetes, smoking status, and alcohol drinking status.

Model 3 was adjusted for variables in model 2 plus high-density lipoprotein cholesterol.

The model for total participants (in which the sexes were combined) was also adjusted for sex.

Crude mortality rate is shown as per 1,000 person-years.

**Figure 1.**
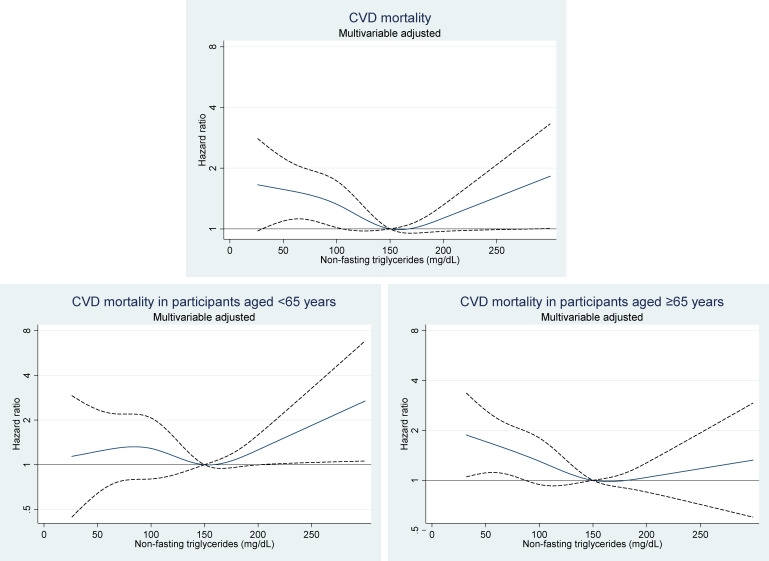
Multivariable-adjusted hazard ratios of non-fasting triglyceride levels for CVD mortality in all participants and in those aged <65 and ≥65 years. Adjusted variables were age, body mass index, total cholesterol, high-density lipoprotein cholesterol, hypertension, diabetes, smoking status, and alcohol drinking status. Non-fasting TG of 150 mg/dL was used as a reference with restricted range of non-fasting TG levels ≤300 mg/dL. CVD, cardiovascular disease; TG, triglyceride.

The crude mortality rates and adjusted HRs for CVD mortality stratified by age <65 and ≥65 years are shown in Table 5, and the spline curves of adjusted HRs are shown in Figure 1. The spline curve for all participants showed a U-shaped association between non-fasting TG levels and CVD mortality. In individuals aged <65 years, the spline curve demonstrated that a non-fasting TG ≥210 mg/dL increased the risk for CVD mortality, while lower levels did not. In contrast, the spline curve for individuals aged ≥65 years demonstrated that a non-fasting TG <80 mg/dL increased the risk for CVD mortality, while higher levels did not. The crude mortality rates and adjusted HRs for ASCVD mortality stratified by age <65 and ≥65 years are shown in Table 6. Lower levels of non-fasting TG ≤59 mg/dL were significantly associated with increased risk for ASCVD mortality in individuals aged ≥65 years. There was a significant interaction between non-fasting TG levels and age group for CVD mortality in men and women overall (*P* for interaction <0.001). For ASCVD mortality, there was no interaction between non-fasting TG levels and age group in men and women overall (*P* for interaction = 0.08).

**Table 5.** Crude mortality rates and hazard ratios for CVD mortality according to non-fasting triglyceride category stratified by age <65 and ≥65 years

	Baseline non-fasting triglyceride level						
	≤59 mg/dL	60–89 mg/dL	90–119 mg/dL	120–149 mg/dL	150–179 mg/dL	180–209 mg/dL	≥210 mg/dL
**CVD death**							
**Age <65 years**							
Men							
Number of participants	176	432	468	311	249	172	430
Person-years	3,238	8,080	8,824	5,864	4,671	3,239	8,085
Number of deaths	8	19	20	10	5	3	24
Crude mortality rate	2.5	2.4	2.3	1.7	1.1	0.9	3
Hazard ratio (95% CI)							
Model 1	2.13 (0.69–6.51)	2.02 (0.75–5.41)	1.88 (0.70–5.01)	1.56 (0.53–4.59)	Ref.	0.91 (0.21–3.83)	2.93 (1.11–7.68)
Model 2	1.86 (0.57–6.08)	2.00 (0.73–5.51)	1.71 (0.63–4.63)	1.45 (0.49–4.29)	Ref.	0.85 (0.20–3.59)	2.57 (0.97–6.82)
Model 3	1.68 (0.49–5.79)	1.88 (0.67–5.29)	1.63 (0.59–4.48)	1.42 (0.48–4.21)	Ref.	0.85 (0.20–3.57)	2.65 (0.99–7.07)

Women							
Number of participants	571	888	634	434	241	145	269
Person-years	11,176	17,310	12,287	8,386	4,657	2,770	5,180
Number of deaths	5	12	13	6	6	5	10
Crude mortality rate	0.4	0.7	1.1	0.7	1.3	1.8	1.9
Hazard ratio (95% CI)							
Model 1	0.81 (0.24–2.70)	0.93 (0.34–2.50)	1.10 (0.42–2.91)	0.56 (0.18–1.74)	Ref.	1.39 (0.42–4.57)	1.42 (0.51–3.91)
Model 2	1.09 (0.31–3.78)	1.17 (0.42–3.20)	1.20 (0.45–3.20)	0.54 (0.17–1.69)	Ref.	1.45 (0.44–4.78)	1.17 (0.42–3.28)
Model 3	1.00 (0.26–3.72)	1.09 (0.38–3.15)	1.15 (0.42–3.13)	0.52 (0.16–1.66)	Ref.	1.46 (0.44–4.81)	1.21 (0.42–3.45)

Total							
Number of participants	747	1,320	1,102	745	490	317	699
Person-years	14,414	25,390	21,111	14,250	9,328	6,009	13,265
Number of deaths	13	31	33	16	11	8	34
Crude mortality rate	0.9	1.2	1.6	1.1	1.2	1.3	2.6
Hazard ratio (95% CI)							
Model 1	1.32 (0.59–2.96)	1.38 (0.69–2.75)	1.42 (0.72–2.82)	0.99 (0.46–2.14)	Ref.	1.16 (0.46–2.88)	2.13 (1.08–4.21)
Model 2	1.43 (0.61–3.33)	1.56 (0.76–3.16)	1.42 (0.71–2.83)	0.93 (0.43–2.02)	Ref.	1.09 (0.44–2.74)	1.83 (0.91–3.65)
Model 3	1.29 (0.53–3.15)	1.46 (0.70–3.03)	1.35 (0.67–2.74)	0.91 (0.42–1.97)	Ref.	1.10 (0.44–2.74)	1.89 (0.94–3.80)

**Age ≥65 years**							
Men							
Number of participants	78	155	127	87	46	41	81
Person-years	855	1,847	1,588	1,169	655	507	1,128
Number of deaths	16	33	27	16	4	13	16
Crude mortality rate	18.7	17.9	17.0	13.7	6.1	25.6	14.2
Hazard ratio (95% CI)							
Model 1	2.70 (0.90–8.11)	2.29 (0.80–6.50)	2.43 (0.85–6.97)	1.97 (0.65–5.91)	Ref.	3.25 (1.05–10.02)	2.07 (0.69–6.22)
Model 2	3.22 (1.02–10.12)	2.53 (0.86–7.40)	2.48 (0.85–7.22)	1.89 (0.62–5.72)	Ref.	3.17 (1.02–9.82)	2.07 (0.68–6.28)
Model 3	4.52 (1.40–14.60)	3.37 (1.13–10.06)	3.02 (1.03–8.87)	2.15 (0.71–6.51)	Ref.	3.17 (1.02–9.79)	1.83 (0.60–5.59)

Women							
Number of participants	53	166	198	116	95	65	103
Person-years	634	2,336	2,793	1,720	1,444	1,074	1,667
Number of deaths	17	37	41	25	16	8	18
Crude mortality rate	26.8	15.8	14.7	14.5	11.1	7.4	10.8
Hazard ratio (95% CI)							
Model 1	2.28 (1.15–4.53)	1.33 (0.73–2.39)	1.36 (0.76–2.42)	1.31 (0.70–2.46)	Ref.	0.68 (0.29–1.59)	1.05 (0.53–2.07)
Model 2	1.97 (0.96–4.05)	1.24 (0.68–2.27)	1.35 (0.75–2.43)	1.29 (0.68–2.43)	Ref.	0.61 (0.26–1.45)	1.10 (0.56–2.18)
Model 3	2.06 (0.98–4.32)	1.30 (0.70–2.42)	1.38 (0.76–2.51)	1.30 (0.69–2.46)	Ref.	0.61 (0.25–1.43)	1.06 (0.52–2.12)

Total							
Number of participants	131	321	325	203	141	106	184
Person-years	1,488	4,182	4,381	2,889	2,099	1,581	2,794
Number of deaths	33	70	68	41	20	21	34
Crude mortality rate	22.2	16.7	15.5	14.2	9.5	13.3	12.2
Hazard ratio (95% CI)							
Model 1	2.16 (1.23–3.79)	1.52 (0.92–2.50)	1.58 (0.96–2.61)	1.43 (0.83–2.44)	Ref.	1.30 (0.70–2.40)	1.29 (0.74–2.24)
Model 2	2.13 (1.18–3.84)	1.49 (0.89–2.50)	1.58 (0.95–2.62)	1.42 (0.83–2.43)	Ref.	1.21 (0.65–2.24)	1.35 (0.77–2.35)
Model 3	2.39 (1.31–4.38)	1.66 (0.98–2.81)	1.69 (1.01–2.82)	1.47 (0.86–2.53)	Ref.	1.19 (0.64–2.21)	1.25 (0.71–2.20)

CI, confidence interval; CVD, cardiovascular disease.

Model 1 was adjusted for age.

Model 2 was adjusted for variables in model 1 plus body mass index, total cholesterol, hypertension, diabetes, smoking status, and alcohol drinking status.

Model 3 was adjusted for variables in model 2 plus high-density lipoprotein cholesterol.

The model for total participants (in which the sexes were combined) was also adjusted for sex.

Crude mortality rate is shown as per 1,000 person-years.

**Table 6.** Crude mortality rates and hazard ratios for ASCVD mortality according to non-fasting triglyceride category stratified by age <65 and ≥65 years

	Baseline non-fasting triglyceride level						
	≤59 mg/dL	60–89 mg/dL	90–119 mg/dL	120–149 mg/dL	150–179 mg/dL	180–209 mg/dL	≥210 mg/dL
**ASCVD death**							
**Age <65 years**							
Men							
Number of participants	176	432	468	311	249	172	430
Person-years	3,238	8,080	8,824	5,864	4,671	3,239	8,085
Number of deaths	4	6	10	5	2	1	9
Crude mortality rate	1.2	0.7	1.1	0.9	0.4	0.3	1.1
Hazard ratio (95% CI)							
Model 1	2.64 (0.48–14.41)	1.57 (0.31–7.79)	2.29 (0.50–10.48)	1.97 (0.38–10.17)	Ref.	0.77 (0.07–8.53)	2.77 (0.59–12.82)
Model 2	2.33 (0.38–14.15)	1.66 (0.32–8.58)	2.09 (0.44–9.84)	1.72 (0.33–9.02)	Ref.	0.68 (0.06–7.60)	2.22 (0.46–10.61)
Model 3	2.54 (0.38–16.93)	1.76 (0.32–9.47)	2.18 (0.45–10.47)	1.76 (0.33–9.26)	Ref.	0.68 (0.06–7.66)	2.17 (0.45–10.45)

Women							
Number of participants	571	888	634	434	241	145	269
Person-years	11,176	17,310	12,287	8,386	4,657	2,770	5,180
Number of deaths	0	4	4	1	2	1	2
Crude mortality rate	0	0.2	0.3	0.1	0.4	0.4	0.4
Hazard ratio (95% CI)							
Model 1	—	0.92	1.01	0.28	Ref.	0.83	0.85
	—	(0.16–5.11)	(0.18–5.55)	(0.02–3.11)		(0.07–9.23)	(0.12–6.06)
Model 2	—	0.89	0.93	0.31	Ref.	0.97	0.93
	—	(0.14–5.45)	(0.16–5.32)	(0.02–3.55)		(0.08–10.87)	(0.12–6.84)
Model 3	—	2.06	1.60	0.44	Ref.	0.93	0.62
	—	(0.28–14.80)	(0.26–9.71)	(0.03–5.15)		(0.08–10.50)	(0.08–4.61)

Total							
Number of participants	747	1,320	1,102	745	490	317	699
Person-years	14,414	25,390	21,111	14,250	9,328	6,009	13,265
Number of deaths	4	10	14	6	4	2	11
Crude mortality rate	0.3	0.4	0.7	0.4	0.4	0.3	0.8
Hazard ratio (95% CI)							
Model 1	1.19 (0.29–4.76)	1.26 (0.39–4.02)	1.64 (0.54–5.00)	1.07 (0.30–3.79)	Ref.	0.79 (0.14–4.32)	1.83 (0.58–5.77)
Model 2	1.04 (0.24–4.48)	1.32 (0.39–4.37)	1.51 (0.48–4.69)	0.93 (0.26–3.35)	Ref.	0.67 (0.12–3.70)	1.58 (0.49–5.12)
Model 3	1.49 (0.32–6.94)	1.68 (0.48–5.83)	1.76 (0.55–5.59)	1.03 (0.28–3.73)	Ref.	0.68 (0.12–3.76)	1.41 (0.43–4.62)

**Age ≥65 years**							
Men							
Number of participants	78	155	127	87	46	41	81
Person-years	855	1,847	1,588	1,169	655	507	1,128
Number of deaths	10	20	13	7	3	6	9
Crude mortality rate	11.7	10.8	8.2	6.0	4.6	11.8	8.0
Hazard ratio (95% CI)							
Model 1	2.40 (0.65–8.75)	1.99 (0.58–6.73)	1.60 (0.45–5.64)	1.20 (0.31–4.65)	Ref.	2.17 (0.54–8.72)	1.61 (0.43–5.95)
Model 2	4.11 (1.06–15.87)	2.92 (0.82–10.31)	1.96 (0.54–7.03)	1.26 (0.32–4.94)	Ref.	1.92 (0.47–7.79)	1.46 (0.38–5.52)
Model 3	5.20 (1.29–20.90)	3.57 (0.98–12.96)	2.24 (0.61–8.16)	1.38 (0.35–5.41)	Ref.	1.92 (0.47–7.76)	1.34 (0.35–5.11)

Women							
Number of participants	53	166	198	116	95	65	103
Person-years	634	2,336	2,793	1,720	1,444	1,074	1,667
Number of deaths	8	17	15	13	7	5	11
Crude mortality rate	12.6	7.3	5.4	7.6	4.8	4.7	6.6
Hazard ratio (95% CI)							
Model 1	2.50 (0.90–6.93)	1.38 (0.57–3.35)	1.12 (0.45–2.76)	1.56 (0.62–3.92)	Ref.	0.96 (0.30–3.04)	1.45 (0.56–3.76)
Model 2	2.04 (0.69–5.99)	1.25 (0.51–3.09)	1.05 (0.42–2.63)	1.49 (0.59–2.43)	Ref.	0.93 (0.29–2.96)	1.51 (0.58–3.92)
Model 3	1.83 (0.60–5.53)	1.12 (0.44–2.87)	1.00 (0.39–2.51)	1.47 (0.58–3.72)	Ref.	0.95 (0.30–3.05)	1.67 (0.62–4.46)

Total							
Number of participants	131	321	325	203	141	106	184
Person-years	1,488	4,182	4,381	2,889	2,099	1,581	2,794
Number of deaths	18	37	28	20	10	11	20
Crude mortality rate	12.1	8.8	6.4	6.9	4.8	7.0	7.2
Hazard ratio (95% CI)							
Model 1	2.33 (1.06–5.08)	1.60 (0.79–3.24)	1.30 (0.63–2.68)	1.37 (0.64–2.95)	Ref.	1.36 (0.58–3.22)	1.48 (0.69–3.18)
Model 2	2.57 (1.13–5.86)	1.70 (0.82–3.51)	1.35 (0.65–2.82)	1.43 (0.66–3.08)	Ref.	1.33 (0.56–3.14)	1.49 (0.69–3.21)
Model 3	2.66 (1.15–6.18)	1.76 (0.83–3.69)	1.38 (0.65–2.90)	1.44 (0.67–3.12)	Ref.	1.32 (0.56–3.13)	1.46 (0.67–3.17)

CI, confidence interval; ASCVD, atherosclerotic cardiovascular disease.

Model 1 was adjusted for age.

Model 2 was adjusted for variables in model 1 plus body mass index, total cholesterol, hypertension, diabetes, smoking status, and alcohol drinking status.

Model 3 was adjusted for variables in model 2 plus high-density lipoprotein cholesterol.

The model for total participants (in which the sexes were combined) was also adjusted for sex.

Crude mortality rate is shown as per 1,000 person-years.

The crude mortality rates and adjusted HRs for all-cause mortality, non-CVD mortality, stroke mortality, and cerebral hemorrhage mortality are shown in eTable 5 and eTable 6. Non-fasting TG levels were not significantly associated with increased risk for all-cause mortality or non-CVD mortality.

## DISCUSSION

In this population-based cohort study in Japan, we found that, compared with non-fasting TG levels of 150–179 mg/dL, non-fasting TG ≥210 mg/dL was significantly associated with increased risk for CVD mortality. Further, lower levels of non-fasting TG were also associated with increased risk for CVD mortality. Similar trends were observed for ASCVD mortality and CHD mortality. In analysis stratified by age, lower levels of non-fasting TG had a stronger impact on increased risk for CVD mortality among individuals aged ≥65 years, while higher levels of non-fasting TG had a stronger impact among those aged <65 years.

For more than three decades, elevated levels of fasting TG have been assessed under the classification of hypertriglyceridemia, for which the proposed cut-off for fasting TG is <150 mg/dL.^17^^–^^19^ Meanwhile, recent studies have suggested that non-fasting TG is a superior predictor for risk of CVD events over fasting TG levels.^5^^,^^6^ For example, the Women’s Health Study reported that, among 27,939 women in the United States, the HR of the highest versus lowest tertile of TG, ≤90 mg/dL versus ≥148 mg/dL, for CVD events was 1.09 (95% CI, 0.85–1.41) for fasting TG and 1.98 (95% CI, 1.21–3.25) for non-fasting TG (≤140 mg/dL versus ≥171 mg/dL).^5^ Levels of non-fasting TG reflect elevated levels of remnant lipoprotein, a subfraction of TG-rich lipoprotein such as chylomicron, after a meal.^7^ Remnant lipoproteins can penetrate the arterial intima, and the cholesterol contained in remnant lipoprotein, remnant cholesterol, accumulates in intimal cells. In contrast, chylomicrons, which are primarily composed of triglycerides, are too large to penetrate the arterial intima.^9^^–^^11^ Accordingly, in the progression of atherosclerosis, remnant cholesterol, rather than elevated TG per se, is thought to be a direct cause of atherosclerosis.^11^ This in turn might suggest that elevated non-fasting TG levels may be a marker of elevated remnant lipoprotein. Consequently, some guidelines have recommended higher cut-off values for non-fasting TG than those for fasting TG for the screening and management of elevated TG.^20^^–^^22^ A scientific statement from the American Heart Association proposed a non-fasting TG cut-off of 200 mg/dL,^20^ the Athens Expert Panel proposed 180 mg/dL,^21^ and a statement from the European Atherosclerosis Society and European Federation of Clinical Chemistry and Laboratory Medicine proposed 175 mg/dL.^22^ The present study showed that a non-fasting TG ≥210 mg/dL significantly increased risk for CVD mortality compared with a non-fasting TG of 150–179 mg/dL, which was associated with the lowest risk. This finding indicates that management of non-fasting TG at levels higher than the cut-off values for fasting TG is also desirable in Japanese populations.

However, the present study also showed that lower levels of non-fasting TG were significantly associated with increased risk for CVD mortality. This result is inconsistent with findings from most previous population-based studies, which have reported that elevated levels of non-fasting and fasting TG are associated with an almost linear increase in risk for CVD events and all-cause mortality.^4^^–^^8^ This discrepancy may have several explanations. First, the present study observed a risk for fatal events only, while previous studies observed a risk for CVD outcomes including both non-fatal and fatal events. This might suggest that individuals with lower levels of non-fasting TG are more likely to die after developing CVD, but do not have a high risk of incident CVD. Indeed, some previous studies have shown that lower levels of casual or fasting TG are significantly associated with elevated severity of disease and increased risk for mortality in patients with coronary artery disease and stroke.^23^^–^^26^ Second, in the present study, there was an almost negative association between non-fasting TG levels and risk for CVD mortality among individuals aged ≥65 years, but not among those aged <65 years. A previous study also reported that TG levels were negatively associated with increased risk for mortality in the oldest population.^27^ The mechanism underlying this “TG paradox” is unclear. However, considering these findings, we speculate that lower levels of non-fasting TG might reflect decreased reserve capacity in aspects, such as nutritional status or health-related factors according to socioeconomic status. Further, the effect of non-fasting TG on CVD mortality might differ according to factors, such as age or the presence or absence of CVD. Dietary TG is absorbed in the small intestine and incorporated into chylomicron, a subfraction of TG-rich lipoprotein, which causes elevated levels of TG after a meal, and then it is carried to the liver.^9^ We speculate that some individuals with low levels of non-fasting TG might take a diet extremely limited on fat or could not eat enough due to any disease. However, our finding of higher risk in low non-fasting TG remained almost unchanged after excluding deaths within first five years of follow-up who were suspected to have had a latent disease at baseline or individuals with low BMI or low TC. Further research is needed in this regard. Considering these findings, management of non-fasting TG will differ among different populations, and should be performed according to population characteristics.

In the present study, we found a weaker association between non-fasting TG levels and CVD mortality among women compared with men, while there was no significant interaction between non-fasting TG and sex for CVD mortality. Previous studies have reported that an atherogenic lipid profile, such as higher levels of low-density lipoprotein cholesterol and total cholesterol, significantly increases risk for CHD in men, but not in women.^28^^–^^30^ A possible reason for this is the difference in cumulative burden from the lipid profile over a lifetime and the incident rates between men and women.^28^ In fact, in the present study, crude mortality for CVD among women was lower than that among men (4.3 per 1,000 person-years for men and 3.0 per 1,000 person-years for women). Accordingly, our findings might also reflect a difference in the effect of the atherogenic lipid profile on the mortality between men and women. On the other hand, a previous study has shown that the association between higher levels of non-fasting TG and CVD mortality is more evident in women.^4^ The participants of this previous study were 20–50 years old, who were younger than participants of the present study. Additionally, the previous study has shown that non-fasting TG was positively linearly associated with CVD mortality. These differences might explain the reason for the different finding in women between the previous study and our study.

The present study had some limitations. First, variables were measured at baseline only; thus, we did not have any information on medications or lifestyle changes during follow-up. Second, causes of death might have been misclassified because death due to CHD might have been included in the ‘heart failure’ category. As a consequence, risk for CHD mortality might have been underestimated.^31^ Finally, non-fatal events were not observed in the present study; thus, risk for the development of CVD including non-fatal events might have been underestimated. Nevertheless, evaluation of risk for fatal events only showed that non-fasting TG levels might have differential impact on fatal CVD events according to population characteristics.

### Conclusion

The present study demonstrated a U-shaped association between non-fasting TG and CVD mortality in a Japanese general population. Among individuals aged ≥65 years, while higher levels of non-fasting TG were not associated with increased risk for CVD mortality, lower levels of non-fasting TG were a predictive marker for CVD mortality. Meanwhile, among those aged <65 years, higher levels of non-fasting TG significantly increased risk for CVD mortality. This indicates that lowering levels of non-fasting TG may prevent CVD deaths among middle-aged individuals, whereas lowering non-fasting TG levels too low may increase risk for CVD in elderly. Further studies including clinical trials are warranted to explore the impact of non-fasting TG levels on CVD mortality in various populations.

## SUPPLEMENTARY MATERIAL

eTable 1. Crude mortality rates and hazard ratios for CVD mortality according to decile category of non-fasting triglyceride

eTable 2. Crude mortality rates and hazard ratios for ASCVD mortality according to decile category of non-fasting triglyceride

eTable 3. Crude mortality rates and hazard ratios for all-cause mortality and non-CVD mortality according to non-fasting triglyceride category

eTable 4. Crude mortality rates and hazard ratios for stroke mortality and cerebral hemorrhage mortality according to non-fasting triglyceride category

eTable 5. Median and interquartile range (mg/dL) according to time since the last meal

eTable 6. The distribution of time since the last meal according to non-fasting triglyceride category
