# Supplementary material for: Relationship Between Non-fasting Triglycerides and Cardiovascular Disease Mortality in a 20-year Follow-up Study of a Japanese General Population: NIPPON DATA90
Source: J Epidemiol. 2022 Jul 5;32(7):303–13. doi: 10.2188/jea.JE20200399 (PMC9189318; doi:10.2188/jea.JE20200399)
Supplement: Supplementary file 1 [file je-32-303-s001.pdf]

eTable 1. Crude mortality rates and hazard ratios for CVD mortality according to decile category of non-fasting triglyceride

	Baseline non-fasting triglyceride level									
	D1 26–55 mg/dL	D2 56–70 mg/dL	D3 71–81 mg/dL	D4 82–93 mg/dL	D5 94–107 mg/dL	D6 108–124 mg/dL	D7 125–145 mg/dL	D8 146–176 mg/dL	D9 177–229 mg/dL	D10 230–1,808 mg/dL
**CVD death**										
Men										
Number of participants	200	250	221	265	280	284	284	323	340	406
Person-years	3,248	4,056	3,813	4,582	4,962	4,937	4,990	5,782	6,092	7,286
Number of deaths	17	22	20	25	21	21	18	13	28	29
Crude mortality rate	5.2	5.4	5.2	5.5	4.2	4.3	3.6	2.2	4.6	4.0
Hazard ratio (95% CI)										
Model 1	1.73 (0.84–3.58)	1.55 (0.78–3.10)	2.12 (1.05–4.27)	1.69 (0.86–3.31)	1.52 (0.76–3.05)	1.63 (0.81–3.26)	1.30 (0.64–2.67)	Ref.	1.95 (1.01–3.76)	1.81 (0.94–3.48)
Model 2	1.86 (0.87–4.01)	1.73 (0.85–3.52)	2.46 (1.21–5.03)	1.82 (0.91–3.63)	1.56 (0.77–3.15)	1.53 (0.76–3.08)	1.31 (0.64–2.68)	Ref.	1.96 (1.01–3.79)	1.90 (0.98–3.69)
Model 3	2.15 (0.97–4.76)	1.94 (0.93–4.01)	1.69 (1.30–5.54)	1.97 (0.98–3.96)	1.67 (0.82–3.39)	1.61 (0.80–3.25)	1.34 (0.65–2.75)	Ref.	1.92 (0.99–3.72)	1.77 (0.90–3.46)

Women										
Number of participants	490	487	445	377	415	404	399	347	340	274
Person-years	9,291	9,071	8,361	6,951	7,506	7,383	7,261	6,350	6,243	5,017
Number of deaths	16	19	17	29	24	22	29	19	24	20
Crude mortality rate	1.7	2.1	2.0	4.2	3.2	3.0	4.0	3.0	3.8	4.0
Hazard ratio (95% CI)										
Model 1	1.92 (0.98–3.74)	1.64 (0.87–3.11)	1.08 (0.56–2.08)	1.69 (0.94–3.01)	1.24 (0.68–2.26)	1.41 (0.76–2.61)	1.48 (0.83–2.64)	Ref.	1.16 (0.63–2.13)	1.33 (0.71–2.50)
Model 2	1.94 (0.96–3.91)	1.63 (0.85–3.12)	1.08 (0.55–2.09)	1.70 (0.94–3.05)	1.22 (0.66–2.24)	1.46 (0.79–2.72)	1.55 (0.86–2.77)	Ref.	1.10 (0.60–2.02)	1.34 (0.71–2.53)
Model 3	1.91 (0.93–3.94)	1.61 (0.82–3.14)	1.06 (0.53–2.11)	1.68 (0.92–3.06)	1.21 (0.65–2.24)	1.45 (0.78–2.72)	1.54 (0.86–2.86)	Ref.	1.10 (0.60–2.03)	1.36 (0.71–2.59)
Total										
Number of participants	690	737	666	642	695	688	683	670	680	680
Person-years	12,539	13,127	12,174	11,533	12,469	12,320	12,251	12,132	12,335	12,304
Number of deaths	33	41	37	54	45	43	47	32	52	49

Crude mortality rate	2.6	3.1	3.0	4.7	3.6	3.5	3.8	2.6	4.2	4.0
Hazard ratio (95% CI)										
Model 1	1.72 (1.05–2.80)	1.49 (0.94–2.37)	1.47 (0.91–2.36)	1.65 (1.06–2.56)	1.35 (0.86–2.13)	1.48 (0.93–2.34)	1.39 (0.89–2.18)	Ref.	1.48 (0.95–2.30)	1.53 (0.98–2.39)
Model 2	1.80 (1.08–3.01)	1.59 (0.99–2.55)	1.57 (0.97–2.55)	1.73 (1.10–2.70)	1.37 (0.87–2.17)	1.46 (0.92–2.31)	1.43 (0.91–2.24)	Ref.	1.42 (0.91–2.22)	1.58 (1.01–2.48)
Model 3	1.93 (1.14–3.29)	1.68 (1.03–2.74)	1.66 (1.02–2.73)	1.80 (1.14–2.83)	1.42 (0.89–2.25)	1.49 (0.94–2.38)	1.45 (0.92–2.28)	Ref.	1.41 (0.90–2.19)	1.52 (0.96–2.40)

CI, confidence interval; CVD, atherosclerotic cardiovascular disease.

Model 1 was adjusted for age.

Model 2 was adjusted for variables in model 1 plus body mass index, total cholesterol, hypertension, diabetes, smoking status, and alcohol drinking status.

Model 3 was adjusted for variables in model 2 plus high-density lipoprotein cholesterol.

The model for total participants (in which sexes were combined) was also adjusted for sex.

Crude mortality rate is shown per 1,000 person-years

eTable 2. Crude mortality rates and hazard ratios for ASCVD mortality according to decile category of non-fasting triglyceride

	Baseline non-fasting triglyceride level									
	D1 26–55 mg/dL	D2 56–70 mg/dL	D3 71–81 mg/dL	D4 82–93 mg/dL	D5 94–107 mg/dL	D6 108–124 mg/dL	D7 125–145 mg/dL	D8 146–176 mg/dL	D9 177–229 mg/dL	D10 230–1,808 mg/dL
**ASCVD death**										
Men										
Number of participants	200	250	221	265	280	284	284	323	340	406
Person-years	3,248	4,056	3,813	4,582	4,962	4,937	4,990	5,782	6,092	7,286
Number of deaths	11	10	10	13	11	11	8	6	14	11
Crude mortality rate	3.4	2.5	2.6	2.8	2.2	2.2	1.6	1.0	2.3	1.5
Hazard ratio (95% CI)										
Model 1	2.40 (0.88–6.51)	1.48 (0.53–4.11)	2.29 (0.83–6.32)	1.84 (0.69–4.85)	1.68 (0.62–4.56)	1.81 (0.67–4.92)	1.23 (0.42–3.56)	Ref.	2.07 (0.79–5.41)	1.46 (0.54–3.96)
Model 2	3.68 (1.27–10.61)	2.10 (0.73–5.98)	3.25 (1.15–9.16)	2.47 (0.91–6.67)	2.00 (0.73–5.50)	1.89 (0.69–5.16)	1.32 (0.45–3.83)	Ref.	2.04 (0.78–5.33)	1.40 (0.51–3.84)
Model 3	4.66 (1.54–14.04)	2.53 (0.86–7.41)	3.74 (1.30–10.68)	2.81 (1.02–7.70)	2.23 (0.80–6.16)	2.04 (0.74–5.60)	1.37 (0.47–3.98)	Ref.	1.97 (0.75–5.17)	1.23 (0.44–3.44)

Women										
Number of participants	490	487	445	377	415	404	399	347	340	274
Person-years	9,291	9,071	8,361	6,951	7,506	7,383	7,261	6,350	6,243	5,017
Number of deaths	7	6	8	11	9	9	12	7	11	10
Crude mortality rate	0.8	0.7	1.0	1.6	1.2	1.2	1.7	1.1	1.8	2.0
Hazard ratio (95% CI)										
Model 1	2.42 (0.84–6.93)	1.46 (0.49–4.35)	1.39 (0.50–3.85)	1.70 (0.66–4.40)	1.21 (0.45–3.27)	1.59 (0.59–4.27)	1.67 (0.65–4.25)	Ref.	1.45 (0.56–3.75)	1.86 (0.70–4.90)
Model 2	1.97 (0.65–5.97)	1.31 (0.43–3.97)	1.24 (0.44–3.48)	1.53 (0.58–4.00)	1.14 (0.42–3.11)	1.57 (0.57–4.26)	1.68 (0.65–4.30)	Ref.	1.44 (0.55–3.73)	1.85 (0.69–4.92)
Model 3	1.99 (0.64–6.21)	1.32 (0.42–4.11)	1.25 (0.43–3.65)	1.54 (0.58–4.10)	1.15 (0.42–3.15)	1.57 (0.57–4.31)	1.68 (0.65–4.31)	Ref.	1.44 (0.55–3.73)	1.84 (0.68–4.99)
Total										
Number of participants	690	737	666	642	695	688	683	670	680	680
Person-years	12,539	13,127	12,174	11,533	12,469	12,320	12,251	12,132	12,335	12,304
Number of deaths	18	16	18	24	20	20	20	13	25	21

Crude mortality rate	1.4	1.2	1.5	2.1	1.6	1.6	1.6	1.1	2.0	1.7
Hazard ratio (95% CI)										
Model 1	2.29 (1.12–4.69)	2.38 (0.66–2.88)	1.78 (0.87–3.65)	1.75 (0.89–3.44)	1.45 (0.72–2.91)	1.68 (0.83–3.38)	1.44 (0.71–2.90)	Ref.	1.73 (0.88–3.39)	1.59 (0.79–3.18)
Model 2	2.56 (1.20–5.48)	1.51 (0.71–3.19)	1.98 (0.95–4.10)	1.90 (0.95–3.77)	1.51 (0.75–3.07)	1.70 (0.84–3.44)	1.52 (0.75–3.08)	Ref.	1.68 (0.86–3.30)	1.61 (0.80–3.23)
Model 3	2.95 (1.34–6.45)	1.69 (0.78–3.65)	2.20 (1.04–4.63)	2.06 (1.02–4.15)	1.62 (0.79–3.30)	1.79 (0.88–3.64)	1.57 (0.77–3.16)	Ref.	1.64 (0.83–3.21)	1.49 (0.73–3.01)

ASCVD, atherosclerotic cardiovascular disease; CI, confidence interval.

Model 1 was adjusted for age.

Model 2 was adjusted for variables in model 1 plus body mass index, total cholesterol, hypertension, diabetes, smoking status, and alcohol drinking status.

Model 3 was adjusted for variables in model 2 plus high-density lipoprotein cholesterol.

The model for total participants (in which sexes were combined) was also adjusted for sex.

Crude mortality rate is shown per 1,000 person-years

eTable 3. Median and interquartile range (mg/dL) according to time since the last meal

	<0.5, n(%)	0.5 to <1, n(%)	1 to <2, n(%)	2 to <3, n(%)	3 to <4, n(%)	4 to <6, n(%)	6 to <8, n(%)
Men	156 [96–201]	138 [94–193]	130 [86–193]	118 [82–177]	116 [85–181]	117 [82–180]	108 [79–153]
Women	103 [75–143]	113 [78–168]	105 [74–150]	105 [73–151]	104 [71–143]	93 [67–133]	85 [62–135]
Total	113 [82–181]	122 [83–176]	112 [77–162]	76 [109–161]	107 [75–159]	103 [73–157]	101 [71–146]

eTable 4. The distribution of time since the last meal according to non-fasting triglyceride category

	Baseline triglyceride level						
Time since the last meal	≤59 mg/dL	60–89 mg/dL	90–119 mg/dL	120–149 mg/dL	150–179 mg/dL	180–209 mg/dL	≥210 mg/dL
**Men (n=2,853)**							
<0.5, n(%)	5 (2.0%)	27 (4.6%)	31 (5.2%)	16 (4.0%)	19 (6.4%)	26 (12.2%)	37 (7.2%)
0.5 to <1, n(%)	8 (3.2%)	27 (4.6%)	27 (4.5%)	30 (7.5%)	16 (5.4%)	18 (8.5%)	28 (5.5%)
1 to <2, n(%)	20 (7.9%)	41 (7.0%)	47 (7.9%)	38 (9.6%)	24 (8.1%)	19 (8.9%)	43 (8.4%)
2 to <3, n(%)	53 (20.9%)	114 (19.4%)	111 (18.7%)	77 (19.4%)	54 (18.3%)	34 (16.0%)	101 (19.8%)
3 to <4, n(%)	44 (17.3%)	95 (16.2%)	99 (16.6%)	67 (16.8%)	46 (15.6%)	35 (16.4%)	84 (16.4%)
4 to <6, n(%)	85 (33.4%)	204 (34.7%)	198 (33.3%)	121 (30.4%)	104 (35.3%)	59 (27.7%)	182 (35.6%)
6 to <8, n(%)	39 (15.3%)	79 (13.5%)	82 (13.8%)	49 (12.3%)	32 (10.9%)	22 (10.3%)	36 (7.1%)
**Women (n=3,978)**							
<0.5, n(%)	24 (3.8%)	67 (6.4%)	57 (6.8%)	30 (5.4%)	17 (5.1%)	14 (6.7%)	23 (6.2%)
0.5 to <1, n(%)	29 (4.7%)	52 (5.0%)	47 (5.7%)	34 (6.2%)	25 (7.4%)	15 (7.1%)	33 (8.9%)
1 to <2, n(%)	62 (9.9%)	109 (10.3%)	93 (11.2%)	68 (12.4%)	47 (14.0%)	22 (10.5%)	38 (12.9%)
2 to <3, n(%)	127 (20.4%)	208 (19.7%)	202 (24.3%)	124 (22.5%)	71 (21.1%)	59 (28.1%)	94 (25.3%)
3 to <4, n(%)	127 (20.4%)	207 (19.6%)	233 (20.3%)	132 (24.0%)	65 (19.3%)	37 (17.6%)	82 (22.0%)
4 to <6, n(%)	198 (31.7%)	335 (31.8%)	233 (28.0%)	129 (23.5%)	88 (26.2%)	51 (24.3%)	79 (21.2%)
6 to <8, n(%)	57 (9.1%)	76 (7.2%)	31 (3.7%)	33 (6.0%)	23 (6.9%)	12 (5.7%)	13 (3.5%)

eTable 5. Crude mortality rates and hazard ratios for all-cause mortality and non-CVD mortality according to non-fasting triglyceride category

	Baseline non-fasting triglyceride level						
	≤59 mg/dL	60–89 mg/dL	90–119 mg/dL	120–149 mg/dL	150–179 mg/dL	180–209 mg/dL	≥210 mg/dL
**All-cause death**							
Men							
Number of participants	254	587	595	398	295	213	511
Person-years	4,093	9,927	10,412	7,033	5,326	3,746	9,213
Number of deaths	101	189	176	107	69	57	133
Crude mortality rate	24.7	19.0	16.9	15.2	13.0	15.2	14.4
Hazard ratio (95% CI)							
Model 1	1.37 (1.01–1.86)	1.04 (0.79–1.38)	1.06 (0.80–1.40)	0.97 (0.71–1.31)	Ref.	1.05 (0.74–1.50)	1.10 (0.82–1.47)
Model 2	1.17 (0.84–1.63)	0.99 (0.75–1.33)	0.97 (0.73–1.29)	0.91 (0.67–1.24)	Ref.	1.03 (0.72–1.46)	1.15 (0.86–1.55)
Model 3	1.21 (0.86–1.71)	1.02 (0.76–1.37)	0.99 (0.74–1.32)	0.92 (0.68–1.26)	Ref.	1.03 (0.72–1.46)	1.14 (0.85–1.54)
Women							
Number of participants	624	1054	832	550	336	210	372
Person-years	11,810	19,645	15,080	10,106	6,102	3,844	6,846
Number of deaths	72	160	179	110	70	46	83
Crude mortality rate	6.1	8.1	11.9	10.9	11.5	12	12.1

Hazard ratio (95% CI)							
Model 1	1.55 (1.11–2.15)	1.20 (0.91–1.59)	1.32 (1.00–1.74)	1.15 (0.85–1.56)	Ref.	0.95 (0.65–1.38)	1.08 (0.78–1.48)
Model 2	1.44 (1.02–2.03)	1.16 (0.87–1.55)	1.29 (0.97–1.71)	1.11 (0.82–1.50)	Ref.	0.86 (0.59–1.25)	1.03 (0.75–1.42)
Model 3	1.47 (1.02–2.10)	1.18 (0.88–1.60)	1.30 (0.98–1.73)	1.12 (0.82–1.51)	Ref.	0.85 (0.59–1.24)	1.02 (0.73–1.41)
Total							
Number of participants	878	1,641	1,427	948	631	423	883
Person-years	15,903	29,572	25,492	17,139	11,428	7,590	16,059
Number of deaths	173	349	355	217	139	103	216
Crude mortality rate	10.9	11.8	13.9	12.7	12.2	13.6	13.5
Hazard ratio (95% CI)							
Model 1	1.43 (1.15–1.79)	1.11 (0.91–1.35)	1.18 (0.97–1.44)	1.05 (0.85–1.30)	Ref.	1.00 (0.78–1.30)	1.10 (0.89–1.36)
Model 2	1.29 (1.02–1.64)	1.07 (0.88–1.32)	1.11 (0.91–1.36)	1.01 (0.81–1.25)	Ref.	0.94 (0.73–1.22)	1.12 (0.90–1.39)
Model 3	1.33 (1.04–1.70)	1.10 (0.89–1.35)	1.13 (0.92–1.38)	1.01 (0.82–1.26)	Ref.	0.94 (0.73–1.22)	1.11 (0.89–1.37)
**Non-CVD death**							
Men							
Number of participants	254	587	595	398	295	213	511
Person-years	4,093	9,927	10,412	7,033	5,326	3,746	9,213
Number of deaths	77	137	129	81	60	41	93
Crude mortality rate	18.8	13.8	12.4	11.5	11.3	10.9	10.1

Hazard ratio (95% CI)							
Model 1	1.21 (0.86–1.70)	0.88 (0.64–1.19)	0.90 (0.66–1.22)	0.84 (0.60–1.18)	Ref.	0.87 (0.59–1.30)	0.88 (0.64–1.23)
Model 2	0.96 (0.66–1.38)	0.80 (0.58–1.11)	0.80 (0.59–1.10)	0.80 (0.57–1.13)	Ref.	0.86 (0.57–1.28)	0.95 (0.68–1.32)
Model 3	0.95 (0.65–1.39)	0.80 (0.57–1.11)	0.80 (0.58–1.10)	0.80 (0.57–1.13)	Ref.	0.86 (0.57–1.28)	0.95 (0.68–1.32)
Women							
Number of participants	624	1054	832	550	336	210	372
Person-years	11,810	19,645	15,080	10,106	6,102	3,844	6,846
Number of deaths	50	111	125	79	48	33	55
Crude mortality rate	4.2	5.7	8.3	7.8	7.9	8.6	8.0
Hazard ratio (95% CI)							
Model 1	1.49 (1.00–2.22)	1.20 (0.85–1.68)	1.34 (0.96–1.86)	1.19 (0.83–1.71)	Ref.	1.00 (0.64–1.56)	1.03 (0.70–1.53)
Model 2	1.36 (0.89–2.06)	1.15 (0.81–1.63)	1.29 (0.92–1.81)	1.13 (0.79–1.62)	Ref.	0.90 (0.57–1.41)	0.97 (0.66–1.44)
Model 3	1.40 (0.91–2.16)	1.18 (0.82–1.70)	1.31 (0.93–1.85)	1.14 (0.79–1.64)	Ref.	0.89 (0.57–1.40)	0.95 (0.64–1.42)
Total							
Number of participants	878	1,641	1,427	948	631	423	883
Person-years	15,903	29,572	25,492	17,139	11,428	7,590	16,059
Number of deaths	127	248	254	160	108	74	148
Crude mortality rate	8.0	8.4	10.0	9.3	9.5	9.7	9.2

Hazard ratio (95% CI)							
Model 1	1.34 (1.03–1.73)	1.01 (0.81–1.27)	1.08 (0.86–1.36)	1.00 (0.78–1.27)	Ref.	0.93 (0.69–1.26)	0.96 (0.75–1.23)
Model 2	1.14 (0.86–1.49)	0.95 (0.75–1.20)	1.00 (0.79–1.26)	0.95 (0.74–1.21)	Ref.	0.88 (0.65–1.18)	0.98 (0.76–1.26)
Model 3	1.14 (0.86–1.52)	0.96 (0.75–1.22)	1.00 (0.79–1.26)	0.95 (0.74–1.21)	Ref.	0.88 (0.65–1.18)	0.98 (0.76–1.26)

CI, confidence interval; Non-CVD, non-cardiovascular disease.

Model 1 was adjusted for age.

Model 2 was adjusted for variables in model 1 plus body mass index, total cholesterol, hypertension, diabetes, smoking status, and alcohol drinking status.

Model 3 was adjusted for variables in model 2 plus high-density lipoprotein cholesterol.

The model for total participants (in which the sexes were combined) was also adjusted for sex.

Crude mortality rate is shown as per 1,000 person-years

eTable 6. Crude mortality rates and hazard ratios for stroke mortality and cerebral hemorrhage mortality according to non-fasting triglyceride category

	Baseline non-fasting triglyceride level						
	≤59 mg/dL	60–89 mg/dL	90–119 mg/dL	120–149 mg/dL	150–179 mg/dL	180–209 mg/dL	≥210 mg/dL
**Stroke death**							
Men							
Number of participants	254	587	595	398	295	213	511
Person-years	4,093	9,927	10,412	7,033	5,326	3,746	9,213
Number of deaths	12	27	16	7	3	5	16
Crude mortality rate	2.9	2.7	1.5	1.0	0.6	1.3	1.7
Hazard ratio (95% CI)							
Model 1	3.61 (1.01–12.84)	3.27 (0.99–10.83)	2.13 (0.62–7.35)	1.40 (0.36–5.43)	Ref.	2.07 (0.49–8.71)	2.99 (0.87–10.29)
Model 2	4.53 (1.21–16.92)	4.05 (1.19–13.75)	2.28 (0.65–7.93)	1.36 (0.35–5.30)	Ref.	2.00 (0.47–8.42)	3.01 (0.87–10.43)
Model 3	3.94 (1.02–15.17)	3.63 (1.04–12.5)	2.12 (0.60–7.43)	1.30 (0.33–5.09)	Ref.	1.98 (0.47–8.33)	3.15 (0.91–10.94)
Women							
Number of participants	624	1054	832	550	336	210	372
Person-years	11,810	19,645	15,080	10,106	6,102	3,844	6,846
Number of deaths	7	17	24	10	13	5	10
Crude mortality rate	0.6	0.9	1.6	1.0	2.1	1.3	1.3

Hazard ratio (95% CI)							
Model 1	0.88 (0.35–2.22)	0.71 (0.34–1.46)	0.95 (0.48–1.87)	0.58 (0.25–1.32)	Ref.	0.55 (0.19–1.55)	0.73 (0.32–1.68)
Model 2	0.69 (0.26–1.84)	0.61 (0.29–1.30)	0.89 (0.44–1.78)	0.56 (0.24–1.28)	Ref.	0.53 (0.18–1.50)	0.79 (0.34–1.82)
Model 3	0.65 (0.23–1.79)	0.58 (0.26–1.28)	0.86 (0.43–1.75)	0.55 (0.24–1.27)	Ref.	0.53 (0.19–1.52)	0.83 (0.35–1.95)
Total							
Number of participants	878	1,641	1,427	948	631	423	883
Person-years	15,903	29,572	25,492	17,139	11,428	7,590	16,059
Number of deaths	19	44	40	17	16	10	26
Crude mortality rate	1.2	1.5	1.6	1.0	1.4	1.3	1.6
Hazard ratio (95% CI)							
Model 1	1.41 (0.72–2.75)	1.20 (0.68–2.14)	1.15 (0.64–2.05)	0.71 (0.36–1.41)	Ref.	0.82 (0.37–1.82)	1.18 (0.63–2.21)
Model 2	1.38 (0.68–2.80)	1.23 (0.68–2.22)	1.14 (0.63–2.06)	0.71 (0.35–1.41)	Ref.	0.78 (0.35–1.72)	1.26 (0.67–2.36)
Model 3	1.24 (0.60–2.59)	1.13 (0.61–2.08)	1.08 (0.59–1.97)	0.69 (0.34–1.37)	Ref.	0.78 (0.35–1.73)	1.33 (0.70–2.51)
**Cerebral hemorrhage death**							
Men							
Number of participants	254	587	595	398	295	213	511
Person-years	4,093	9,927	10,412	7,033	5,326	3,746	9,213
Number of deaths	3	7	3	3	1	2	3

Crude mortality rate	0.7	0.7	0.3	0.4	0.2	0.5	0.3
Hazard ratio (95% CI)							
Model 1	3.36 (0.34–32.41)	3.17 (0.38–25.89)	1.38 (0.14–13.33)	2.03 (0.21–19.60)	Ref.	2.81 (0.25–31.01)	1.76 (0.18–17.00)
Model 2	3.38 (0.31–36.51)	3.36 (0.39–28.97)	1.28 (0.12–12.80)	1.77 (0.18–17.30)	Ref.	2.85 (0.25–31.64)	1.52 (0.15–14.90)
Model 3	3.62 (0.31–42.00)	3.52 (0.39–31.51)	1.32 (0.13–13.50)	1.80 (0.18–17.63)	Ref.	2.85 (0.25–31.67)	1.49 (0.15–14.70)
Women							
Number of participants	624	1054	832	550	336	210	372
Person-years	11,810	19,645	15,080	10,106	6,102	3,844	6,846
Number of deaths	2	4	3	3	3	0	3
Crude mortality rate	0.2	0.2	0.2	0.3	0.5	0.0	0.4
Hazard ratio (95% CI)							
Model 1	1.05 (0.17–6.42)	0.71 (0.15–3.19)	0.53 (0.10–2.63)	0.76 (0.15–3.81)	Ref.	–	0.92 (0.18–4.60)
Model 2	0.66 (0.09–4.51)	0.54 (0.11–2.59)	0.39 (0.07–2.06)	0.71 (0.14–3.57)	Ref.	–	1.04 (0.20–5.20)
Model 3	0.47 (0.06–3.55)	0.41 (0.07–2.13)	0.34 (0.06–1.81)	0.67 (0.13–3.37)	Ref.	–	1.33 (0.25–7.07)
Total							
Number of participants	878	1,641	1,427	948	631	423	883
Person-years	15,903	29,572	25,492	17,139	11,428	7,590	16,059
Number of deaths	5	11	6	6	4	2	6
Crude mortality rate	0.3	0.4	0.2	0.4	0.4	0.3	0.4

Hazard ratio (95% CI)							
Model 1	1.42 (0.38–5.33)	1.24 (0.39–3.92)	0.70 (0.19–2.51)	1.02 (0.28–3.62)	Ref.	0.70 (0.12–3.84)	1.05 (0.29–3.74)
Model 2	1.16 (0.28–4.71)	1.15 (0.35–3.75)	0.62 (0.17–2.26)	0.93 (0.26–3.35)	Ref.	0.66 (0.12–3.66)	1.11 (0.31–3.99)
Model 3	1.06 (0.24–4.53)	1.07 (0.31–3.63)	0.59 (0.16–2.19)	0.91 (0.25–3.29)	Ref.	0.67 (0.12–3.68)	1.16 (0.32–4.24)

CI, confidence interval.

Model 1 was adjusted for age.

Model 2 was adjusted for variables in model 1 plus body mass index, total cholesterol, hypertension, diabetes, smoking status, and alcohol drinking status.

Model 3 was adjusted for variables in model 2 plus high-density lipoprotein cholesterol.

The model for total participants (in which the sexes were combined) was also adjusted for sex.

Crude mortality rate is shown as per 1,000 person-years
